# Scars of War: Unveiling the Psychiatric Consequences of Conflict and Trauma

**DOI:** 10.1192/j.eurpsy.2025.299

**Published:** 2025-08-26

**Authors:** A. Sousa, J. L. Guimarães, M. F. Fernandes

**Affiliations:** 1 Serviço de Psiquiatria e Saúde Mental, ULS Castelo Branco, Castelo Branco; 2 C.R.I. de Saúde Mental, Unidade Local de Saúde de Castelo Branco, Branco, Portugal

## Abstract

**Introduction:**

War and conflict have significant long-term effects on mental health, affecting both civilians and military personnel. Exposure to armed conflict greatly increases the risk of psychiatric disorders such as PTSD, depression, and anxiety. These effects are not limited to the immediate aftermath but can persist for years, even across generations. Vulnerable populations, including children, women, and refugees, are particularly at risk, facing compounded mental health challenges due to prolonged exposure to trauma and limited access to support.

**Objectives:**

This poster aims to:

1. Explore the psychiatric consequences of war and conflict.
2. Investigate the development of PTSD, depression, and anxiety in conflict-affected populations.
3. Assess the role of transgenerational trauma transmission and its epigenetic effects.
4. Highlight the role of resilience and psychosocial support in mitigating these effects.

**Methods:**

Data were drawn from various studies examining populations affected by conflict across different regions. Key metrics included the prevalence of PTSD, depression, and anxiety, and the impact of traumatic experiences. The role of social support and resilience factors in mitigating psychiatric outcomes was also analyzed.

**Results:**

The findings consistently demonstrate high rates of PTSD and other psychiatric disorders in individuals exposed to conflict. Mental health impacts are often prolonged, with many individuals showing persistent symptoms years after the initial trauma. Additionally, research on epigenetic changes suggests that trauma can be passed down to future generations, potentially affecting their mental health. However, protective factors such as strong social support and community resilience were found to buffer the negative effects of trauma in some cases.

**Conclusions:**

War and conflict have profound and lasting psychiatric consequences. The prevalence of mental health disorders is significant among affected populations, and these effects can be transmitted across generations. Interventions should focus on providing timely mental health care, building resilience, and addressing the broader societal impacts of trauma. Early identification and support are critical to reducing the long-term mental health burden in conflict-affected areas.

**Disclosure of Interest:**

None Declared

